# Indiana

**Published:** 1901-05

**Authors:** 

**Affiliations:** Chairman


### INDIANA.

A bill for an act entitled "An Act to Define Veterinary Medicine and Surgery, and Regulating the Practice of Veterinary Surgery or any branch thereof in the State of Indiana."—Engrossed Senate Bill No. 291.

*Be it enacted by the General Assembly of the State of Indiana:*

Section 1. That the practice of veterinary medicine or surgery within the meaning of this act shall be any act or operation, the prescribing or giving of medicine for the relief of diseases, injury or accident, for the correction of habit, defective act, deformity or vice, spaying, castration, obstetrics, and dentistry upon any domestic animal.

Sec. 2. The right to use the degree or title veterinarian, veterinary surgeon, doctor of veterinary medicine or surgery, doctor of comparative medicine, or any derivative thereof, shall be limited to graduates of reputable veterinary colleges.

Sec. 3. Any person practising veterinary medicine or surgery and having a degree from a reputable veterinary college, shall be exempt from jury duty and shall be entitled to expert witness fees when summoned or required to testify in any civil action when such testimony relates to matters connected with the veterinary profession.

Sec. 4. It shall be unlawful for any person to use any degree or title pertaining to the practice of veterinary medicine or surgery, other than as provided in section two of this act, and any person so doing be subject to a fine of not less than twenty dollars nor more than fifty dollars.

Sec. 5. It shall be unlawful for any person to practice veterinary medicine or surgery, or any branch thereof, who is not a graduate of a reputable veterinary college : *Provided*, That nothing in this act shall apply to persons who have practised veterinary medicine or surgery in this State for five consecutive years as a livelihood, immediately preceding the passage of this act, as certified to by five freeholders before the county clerk where he resides, nor for the operations of castration, spaying, dehorning, or assistance rendered in emergencies, nor shall it apply to persons practising upon their own animals. Any person so doing shall be subject to the same penalties as provided in section four.

Sec. 6. All persons qualified under this act to practice veterinary medicine and surgery, shall have the same recognition in prescription work as now accorded to regular practitioners of medicine, by druggists and pharmacists.

Sec. 7. All persons desiring to practice veterinary medicine and surgery in the State of Indiana, shall, within ninety days after the taking effect of this act, file with the clerk of the court of the county in which the applicant resides, the necessary evidence as to the qualifications to entitle them to practice according to the provisions of this act. Upon filing such evidence the clerk shall issue to such applicant a certificate to practice in accordance with the provisions of this act, in any county in the State of Indiana, such blank certificates to be furnished by the State Board of Health. The county clerk shall keep a record of all persons in each county qualified to practice according to the provisions of this act. For such services the clerk shall receive from each applicant the sum of one dollar for such registration.

Sec. 8. All laws and parts of laws in conflict with this act are hereby repealed.

Mr. Speaker: Your Committee on Medicine, Health and Vital Statistics, to which was referred Engrossed Senate Bill No. 291, entitled a bill for an act entitled "An Act to Define Veterinary Medicine and Surgery, and Regulating the Practice of Veterinary Surgery or any branch thereof in the State of Indiana," introduced by Mr. Keyes, has had the same under consideration, and begs leave to report the same back to the House with the recommendation that said bill be amended by striking out in section two the words, "graduates of reputable veterinary colleges," and insert in lieu thereof the words, "to those holding a license to practice under this act," and by striking out of section three the words "and shall be entitled to expert witness fees when summoned or required to testify in any civil action when such testimony relates to matters connected with the veterinary profession," and that when so amended that the bill do pass.

VAN FLEET.
Chairman.

